# Effects of Reproductive Status, Social Rank, Sex and Group Size on Vigilance Patterns in Przewalski's Gazelle

**DOI:** 10.1371/journal.pone.0032607

**Published:** 2012-02-28

**Authors:** Chunlin Li, Zhigang Jiang, Linlin Li, Zhongqiu Li, Hongxia Fang, Chunwang Li, Guy Beauchamp

**Affiliations:** 1 Key Laboratory of Animal Ecology and Conservation Biology, Institute of Zoology, Chinese Academy of Sciences, Beijing, China; 2 Graduate University of the Chinese Academy of Sciences, Beijing, China; 3 College of Life Science, Nanjing University, Nanjing, China; 4 Faculty of Veterinary Medicine, University of Montréal, Québec, Canada; University of Manitoba, Canada

## Abstract

**Background:**

Quantifying vigilance and exploring the underlying mechanisms has been the subject of numerous studies. Less attention has focused on the complex interplay between contributing factors such as reproductive status, social rank, sex and group size. Reproductive status and social rank are of particular interest due to their association with mating behavior. Mating activities in rutting season may interfere with typical patterns of vigilance and possibly interact with social rank. In addition, balancing the tradeoff between vigilance and life maintenance may represent a challenge for gregarious ungulate species rutting under harsh winter conditions. We studied vigilance patterns in the endangered Przewalski's gazelle (*Procapra przewalskii*) during both the rutting and non-rutting seasons to examine these issues.

**Methodology/Principal Findings:**

Field observations were carried out with focal sampling during rutting and non-rutting season in 2008–2009. Results indicated a complex interplay between reproductive status, social rank, sex and group size in determining vigilance in this species. Vigilance decreased with group size in female but not in male gazelles. Males scanned more frequently and thus spent more time vigilant than females. Compared to non-rutting season, gazelles increased time spent scanning at the expense of bedding in rutting season. During the rutting season, territorial males spent a large proportion of time on rutting activities and were less vigilant than non-territorial males. Although territorial males may share collective risk detection with harem females, we suggest that they are probably more vulnerable to predation because they seemed reluctant to leave rut stands under threats.

**Conclusions/Significance:**

Vigilance behavior in Przewalski's gazelle was significantly affected by reproductive status, social rank, sex, group size and their complex interactions. These findings shed light on the mechanisms underlying vigilance patterns and the tradeoff between vigilance and other crucial activities.

## Introduction

Vigilance in animals may reduce the likelihood of being attacked and thus increase fitness by improving the ability to survive, obtain resources, reproduce and protect offspring [1], [2]. However, vigilance behavior is often performed at the expense of other fitness-enhancing activities crucial for life maintenance and reproduction, such as foraging, resting and mating [3], [4]. Quantifying vigilance patterns and exploring underlying influencing factors help us to understand how animals respond to potential threats and balance the tradeoff between vigilance and other crucial activities [5].

Factors influencing vigilance behavior have been studied in a wide variety of birds and mammals [2]. In ungulates, vigilance is influenced by sex [6], [7], level of predation risk [8], [9], group size [10], [11] and position in the herd [8]. While not universal [12], many studies have detected a decrease in individual vigilance in larger groups, which is usually explained by the many-eyes effect, risk dilution or scramble competition [13], [14], [15]. The first two mechanisms, related to anti-predatory vigilance, imply that the presence of more companions can lead to better threat detection and a simple dilution of risk upon attack. The third hypothesis argues that increased competition for limited resources in larger groups forces individuals to relax vigilance to increase their relative shares of limited resources [16].

Season is an important factor influencing activity patterns [17], [18], [19]. Most studies examining the effect of season on vigilance have focused on the calving season, and have found that mothers with young spend more time vigilant [20], [21]. Rutting season is also a critical period where animals spend considerable time and energy in mating activities [22]. Vigilance patterns may differ with seasonal changes in reproductive status [23], [24]. Thus far, however, studies concentrating on the effect of reproductive status and its interaction with other factors have been limited. Particularly, little attention has been paid to ungulates that rut under harsh winter conditions. For such species, balancing the tradeoff between vigilance and life maintenance while rutting could be a challenge. Given the time demands for rutting, especially for males, and the fact that food is often of poor quality in winter, maintaining adequate levels of vigilance may be quite difficult without compromising body condition [25], [26]. Investigation of vigilance patterns under such conditions could help to understand strategies to allocate limited time to vigilance under predation risk.

During the rutting season, animals of different social ranks vary in mating tactics and in their allocation of time and energy to daily activities [27], [28]. Vigilance patterns may thus differ in animals of different social ranks [8]. A survey of the literature examining the effect of social rank on vigilance behavior in rutting ungulates reveals contradictory findings [10], [29], [30], [31]. For instance, territorial males in impala (*Aepyceros melampus*) allocated more time to vigilance against intruding rivals [10]. In elk (*Cervus elaphus*), however, rutting bulls engaged in courtship spent little time vigilant [30]. Social rank is typically used to account for differences in vigilance between dominant and subordinate individuals [10], [30]. However, discrepancy in the results so far may reflect interactions between social rank and species-specific mating systems, which suggests that more empirical studies are needed to investigate the effect of social rank on vigilance behavior during the rutting season.

We used Przewalski's gazelle (*Procapra przewalskii*) as a model species to investigate the effects of reproductive status, social rank, sex and group size on vigilance patterns in ungulates. Mating system of Przewalski's gazelle is characterized as the female traffic version of the hotspot hypothesis [32]. After fights among male gazelles prior to winter rutting, each winner herds a harem of females that roam along a relatively fixed daily travel route. Losers forage apart with occasional challenges to dominant males resulting in sporadic mating chances. This type of mating system provides an ideal model to investigate behavioral strategies, especially the tradeoff between essential activities in socially foraging animals. Li et al. [33] and Li et al. [34] have found that vigilance in Przewalski's gazelle was influenced by group size, distance to human infrastructure and level of predation risk, and that these effects also varied between the sexes. Here, we intended to determine: 1) whether vigilance patterns differ between the rutting and non-rutting season; 2) if so, whether the effect of reproductive status differs between the sexes; and 3) whether and how social rank influences vigilance behavior during the rutting season. We predicted that gazelles would be more vigilant in rutting season because of increased demands for monitoring conspecifics with potentially different outcomes for males and females and for individuals of different social rank. We also attempted to understand how gazelles balance the tradeoff between vigilance, feeding, bedding and rutting activities based on time allocated to these essential behaviors.

## Methods

### Ethics Statement

We adhered to the “Guidelines for the use of animals in research” published in *Animal Behaviour*. Our research protocols have been approved by the Chinese Wildlife Management Authority. The study was observational involving no cruelty to animals and thus no review from the ethnic committee was required in China. All the work was carried out under the Wildlife Protection Law of the People's Republic of China.

### Study Site and Species

Field observations were carried out using one population of Przewalski's gazelle in Shengge (37°27′48″ N, 98°33′45″ E) along the Upper Buha River Valley, located in the north-east of the Qinghai-Tibetan Plateau, China. Elevation in the distribution area of this population ranges from 3,500 m to 4,000 m above sea level. The region has an inland plateau semi-arid climate with dry, cold and long winters and a short frost-free period. Mean annual temperature is −1.5°C with the lowest record of −40°C. Annual precipitation is 330∼500 mm with mean evaporation of 1,300∼2,000 mm. Dominant vegetation type is alpine meadow with shrubs along the Buha River Valley. The main predator is wolf (*Canis lupus*) with an estimated population of ten individuals in this area. Tibetan fox (*Vulpes ferrilata*) is also common, contributing to some calf mortality [34]. Human disturbance is rare.

Przewalski's gazelle is an endangered species endemic to the Qinghai-Tibetan Plateau [35], [36], [37]. Since the 1950 s, the gazelle's population has significantly declined and is now restricted to several isolated areas around the Qinghai Lake in the north-east of the plateau [38], [39]. The overall population size of the gazelle is estimated at 1,200∼1,600 with approximately 100 adult gazelles in Shengge [40]. Sympatric Tibetan gazelle (*Procapra picticaudata*) occasionally forages with Przewalski's gazelle in the Shengge area [34], [41]. Rutting of Przewalski's gazelle lasts from late December to mid-January [36]. During the rutting season, in the winter 2008–2009 when this study was carried out, two types of groups were found: numerous non-territorial male groups and nine relatively stable reproductive groups composed of one territorial male and harem females. During the non-rutting season in the summer 2009, the two sexes mainly foraged apart (∼20 groups), which is referred to as sexual segregation [17], [42].

### Behavioral Observation

Field observations were carried out on sunny days from December 24, 2008 to January 15, 2009 (rutting season of Przewalski's gazelle) and from June 5 to June 30, 2009 (non-rutting season). During field work, we used focal sampling [43] to record behaviors from sunrise to sunset, i.e. 07:00 to 20:00 in summer and 08:30 to 18:00 in winter.

We selected groups of Przewalski's gazelle for behavioral observation as they were encountered along a fixed route. We defined a focal group as a collection of individuals all occurring within 50 m of one another [34]. Group members were mentally numbered from left to right according to their location in the group. Using a random number generator, one focal individual was then selected among those present. In order to minimize the likelihood of pseudo-replication, no group was observed more than once on the same day, and we only selected one focal adult individual in a focal group [34], [44]. With this random procedure, we estimated that all individuals within a category had the same chances of being sampled. Since it was not possible to mark individuals, the same individuals may have been watched on different days. However, this probably did not occur very often due to the large number of gazelles in the area. In addition, intervening changes in group size and spatial position within groups between observation days created very different contingencies for any successive observation on the same subjects. Therefore, it is reasonable to assume that pseudo-replication is not a major issue here.

We defined five categories of individuals with respect to reproductive status, sex and social rank: females, non-territorial males and territorial males in rutting season and females and males in non-rutting season (Table 1). Social rank was specific to males (territorial and non-territorial males) during the rutting season. Territorial males were easily recognized by morphological characteristics (denser neck color, prominent larynx and exposed penis), rutting behaviors (herding and mounting females, mating roar, frequently marking rut stands with urine and chasing away intruding males) and relatively stable rut stands along female travel routes. Non-territorial males forage alone or in small single-sex groups away from territorial males and harem females [32], [45]. Li et al. [34] found that mother gazelles with calves were generally more vigilant than females without calves. Therefore, to eliminate the effect of attending calves in females, we did not select mother gazelles as focal individuals in our field observations. This choice also makes the comparison of females between seasons simpler. Subadults were pooled with adults as they reach adult size in their first winter and were not practically distinguishable from adults in the field [34]. Finally, we only considered single-species herds as focal groups to avoid the effect of “additional eyes” from different species [46], [47].

**Table 1:** Summary of focal observation samples as a function of individual status in Przewalski's gazelle.

	Number of observations	Total observation time (min)	Average group size	Range of group size
Rutting season				
Females	87	2248.7	10.1 (±0.8)	2–34
Non-territorial males	68	1399.1	1.1 (±0.1)	1–4
Territorial males	81	2068.6	9.6 (±0.6)	2–34
Non-rutting season				
Females	94	2428.6	6.2 (±0.4)	1–20
Males	69	1719.4	3.1 (±0.3)	1–9

For each observation session, we recorded behavior, season, date, time, group size, herd composition, sex and social rank of the focal gazelle. We recorded six behavioral states: feeding, bedding, moving, vigilance, rutting activity and other behaviors. Feeding was defined as grazing or short bout of searching between grazing bouts with the head held below the shoulders. Bedding referred to rest and rumination while sitting on the ground. Moving was defined as walking or trotting with the head held above the horizontal plane without foraging. A gazelle was considered vigilant when it was standing with the head above shoulder level and scanning. Obvious alert scanning during other activities was also regarded as vigilance. Rutting activities in males included herding and guarding harem females, displaying, mounting, mating and marking rut stands with urine as well as chasing away intruders of the same sex. Females showed fewer and simpler rutting activities, including tail wagging and accepting mating [45]. Other behaviors included behaviors that were not listed in the above categories, such as defecating, sneezing and scratching [34]. For females and territorial males during the rutting season, we defined group size as the number of adults in the mixed-sex group including the dominant male and harem females. In the non-rutting season, group size was the total number of adult individuals in a group.

Behaviors were dictated as they occurred on an Mp3 digital recording pen inside an off-road vehicle using binoculars (8×42) or a telescope (20–60×63). Field observations have shown previously that Przewalski's gazelle habituate to parked vehicles, as found in other ungulates [48]. Vehicles moving slowly could often approach gazelles within 100 m with no apparent reaction. To avoid possible disturbances from the vehicle, we slowly drove the vehicle toward the target group and stopped 150∼200 m away. Furthermore, we waited inside the vehicle several minutes prior to recording behaviors. We stopped a focal observation when the focal individual was out of sight, when the focal group composition changed or human disturbances occurred. Field observation and recording task was carried out by the same person.

### Statistical Analysis

Information from Mp3 records was transcribed into timed sequences of behavioral states in EXCEL spreadsheet with the ETHOM software version 1.0 (available at http://web.nchu.edu.tw/~htshih/ethom/intro_c.htm) [49]. To reduce stochastic variation due to short sampling sessions and to increase data reliability and representativeness of the overall time budget in Przewalski's gazelle, observations shorter than five minutes were discarded, as was done in other studies [8], [44]. To obtain percentages of time spent in different activities, the total amount of time spent in one activity during a focal observation was divided by the duration of the focal observation. In addition, we calculated the number of vigilance bouts, which represents the number of transitions to a vigilant state in a focal observation. Mean scan duration represented the amount of time spent vigilant divided by the number of vigilant bouts in one focal observation. Scan frequency represented the number of vigilant bouts divided by focal observation duration and expressed as frequency per min. Prior to statistical analysis, data were tested for normality with the Shapiro-Wilk Test. Percentages were arcsine-square-root transformed and mean scan durations were log_10_ transformed to meet assumptions of normality and homogeneity of variances. All statistical analyses were carried out with SAS v. 8.1 (SAS Institute Inc., Cary, NC, USA) with a level of statistical significance set at 0.05. Data below are shown as mean (± SE).

For the analysis of percentage time spent scanning and mean scan duration (the response variables), a linear model (GLM, PROC GLM in SAS) was used. We first considered the effects of reproductive status (two levels: rutting or non-rutting), sex (female or male), group size (continuous factor) and all interactions of these explanatory variables. Social rank (two levels: territorial or non-territorial) was specific to males during the rutting season. Therefore, we fitted an independent GLM to test the effect of social rank and group size and the interaction between the two, using data from territorial and non-territorial males during the rutting season. Non-significant effects (p>0.05) were excluded in the final models by backward elimination. We included time of day (three levels: before 11:00, 11:00–15:00, after 15:00) as a categorical factor in an earlier analysis but found no significant effect (F_2,396_ = 0.10, t = 0.32, p = 0.748 for percentage time spent scanning; F_2,396_ = 0.20, t = −0.45, p = 0.654 for scan duration) and thus did not consider this factor further.

A generalized linear model (PROC GENMOD in SAS), with a negative-binomial error structure and a logarithmic link function, was fitted to the heavily right-skewed scan frequency data. We used number of scanning bouts in focal observations as the response variable and included the natural logarithm of observation time as an offset because focal observation duration varied among individuals. The same fixed factors as used in the linear model were included in the model. Time of day was also removed due to its non-significant effect (χ^2^ = 1.02, p = 0.312, df = 2). The ratios of the deviance to degrees of freedom were approximately equal to one, indicating good fitness of the models to the data.

Pearson's correlation was used to test the relationship between percentage time spent scanning, feeding, bedding and rutting.

## Results

A total of 399 focal observations, amounting to 9,864 min, were collected over the two seasons (Table 1). Duration of focal observations ranged from 5.0 min to 32.9 min with an average of 24.7 min (±0.3).

### Percentage time spent scanning

**Figure 1:**
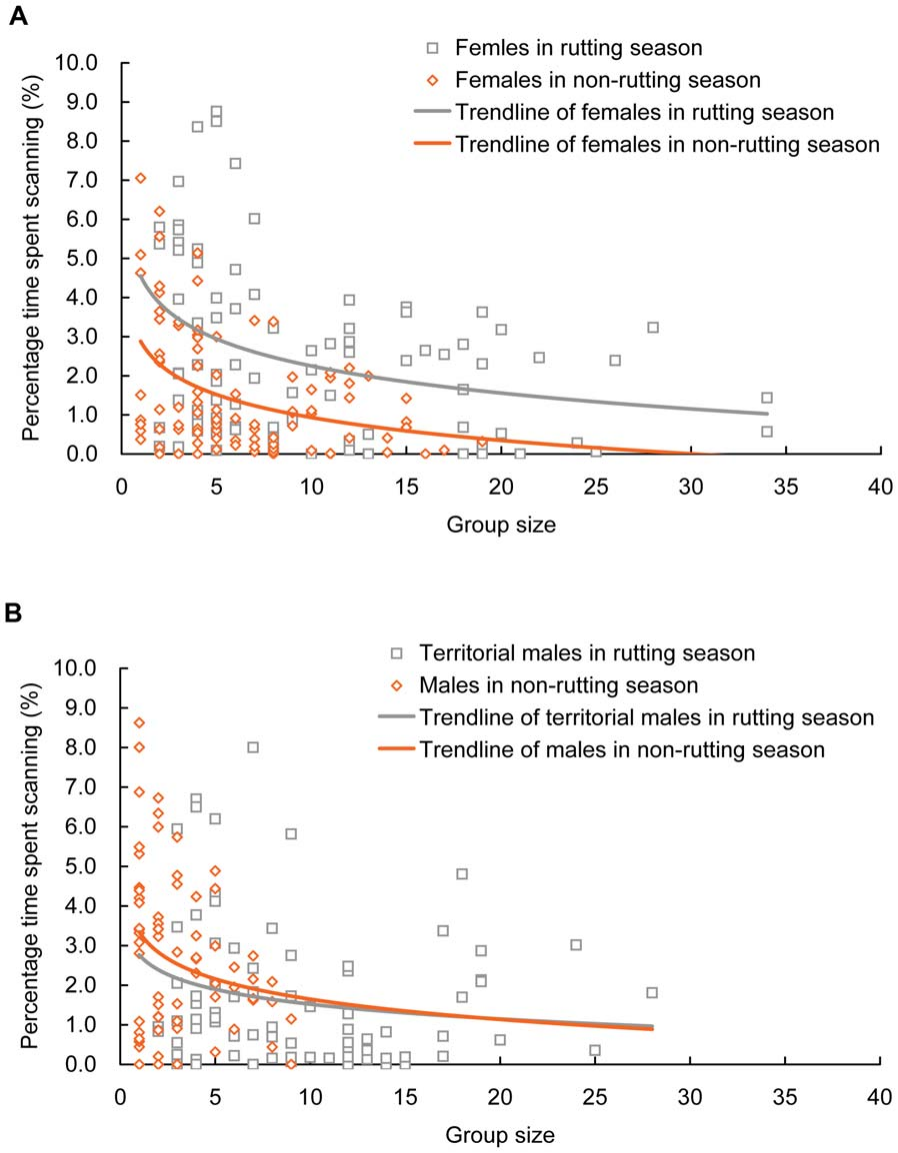
Relationship between percentage time spent scanning and group size in female A) and male B) Przewalski's gazelle. Non-territorial males in rutting season often grazed alone. Thus, the relationship between percentage time spent vigilant, which averaged 7.0%±0.9% and peaked at 33.4%, and group size in non-territorial males is not displayed.

Percentage time spent scanning averaged 2.9% (±0.2%) and ranged from 0 to 33.4%. The final model without social rank revealed a significant effect of sex, reproductive status, group size and an interaction between sex and group size. The interaction between sex and group size indicated a negative effect of group size in females (β = −0.002±0.001, t = −2.72, p = 0.007) but not in males (β = −0.003±0.002, t = −2.04, p = 0.113) (Fig. 1). Males (3.6%±0.3%) spent more time scanning than females (2.0%±0.1%). Percentage time spent scanning increased in rutting (3.5%±0.3%) compared to non-rutting season (2.1%±0.1%). The independent model to test the effect of social rank indicated that non-territorial males (7.0%±0.9%) spent more time scanning than territorial males (1.6%±0.2%) during the rutting season (Table S1).

### Scan duration

Scan duration averaged 10.3 s (±0.6) and ranged from 0 to 112.8 s. The final model without social rank revealed a significant effect of sex and group size. Scan duration decreased with group size (β = −0.008±0.003, t = −2.25, p = 0.025). Mean scan duration in females (11.3±1.0 s) was longer than in males (9.6±0.6 s). There was no significant difference between rutting (10.4±0.8 s) and non-rutting season (10.3±0.8). The independent model to test the effect of social rank indicated that there was no significant difference between territorial (9.0±0.8 s) and non-territorial males (9.6±0.6 s) during the rutting season (Table S2).

### Scan frequency

Scan frequency averaged 0.17 min^−1^ (±0.01) and ranged from 0 to 1.36 min^−1^. The final model without social rank revealed a significant effect of sex and an interaction between sex and group size. There was no overall significant effect of group size on scan frequency. The interaction between sex and group size indicated that scan frequency decreased with group size in females (β = −0.080±0.013, χ^2^ = 38.92, p<0.001) but not in males (β = −0.015±0.009, χ^2^ = 2.56, p = 0.110). Scan frequency was significantly higher in males (0.22±0.02 min^−1^) than in females (0.12±0.01 min^−1^). The independent model to test the effect of social rank indicated that scan frequency in non-territorial males (0.41±0.04 min^−1^) was significantly higher than in territorial males (0.11±0.01 min^−1^) during the rutting season (Table S3).

### Relationship between time spent scanning, feeding, bedding and rutting

Time spent scanning was relatively small in Przewalski's gazelle. Gazelles devoted a large proportion of time to feeding and bedding, the other two main activities (Table 2). During the rutting season, percentage time spent scanning was negatively correlated with bedding (r = −0.34, p = 0.001, N = 87 in females; r = −0.23, p = 0.038, N = 81 in territorial males; r = −0.61, p<0.001, N = 68 in non-territorial males) but positively correlated with feeding (r = 0.24, p = 0.027, N = 87 in females; r = 0.26, p = 0.020, N = 81 in territorial males; r = 0.29, p = 0.018, N = 68 in non-territorial males). No significant relationship was found between percentage time spent scanning and rutting in territorial males (r = −0.09, p = 0.422, N = 81). Rutting activities did not occur frequently in non-territorial males and in females, preventing us for calculating the correlation between scanning and rutting in these animals.

**Table 2:** Breakdown of time spent (%) in different activities in different categories of Przewalski's gazelle.

	Vigilance	Feeding	Bedding	Rutting	Moving	Otherbehaviors
Rutting season						
Females	2.5 (±0.2)	73.3 (±2.7)	14.5 (±2.8)	0.1 (±0.0)	8.7 (±1.0)	0.9 (±0.2)
Non-territorial males	7.0 (±0.9)	44.5 (±3.7)	21.3 (±4.3)	0.4 (±0.1)	22.0 (±2.6)	4.8 (±1.2)
Territorial males	1.6 (±0.2)	32.1 (±2.8)	21.6 (±2.7)	18.1 (±1.6)	20.9 (±1.7)	5.7 (±0.8)
Non-rutting season						
Females	1.6 (±0.2)	58.0 (±3.0)	24.3 (±2.8)	0	13.0 (±1.2)	3.1 (±0.4)
Males	2.7 (±0.3)	31.7 (±3.5)	41.7 (±4.2)	0	20.6 (±2.7)	3.3 (±0.6)

During the non-rutting season, a negative correlation was found between percentage time spent scanning and feeding (r = −0.32, p = 0.002, N = 94 in females; r = −0.21, p = 0.080, N = 69 in males) but a positive correlation was documented between scanning and bedding (r = 0.21, p = 0.042, N = 94 in females; r = 0.29, p = 0.015, N = 69 in males).

## Discussion

Overall, the results illustrate that vigilance in Przewalski's gazelle reflects a complex interplay between reproductive status, social rank, sex and group size. Changes in time spent vigilant were caused by changes in both scan frequency and scan duration. A recent meta-analysis in birds found that the effect size related to scan frequency was usually larger than that for scan duration [12]. A similar meta-analysis is lacking in mammals so that it is difficult to judge whether the pattern documented in gazelles here is common in other mammals. In the following, we focus on time spent vigilant as a proxy for vigilance since the effect of group size was consistent among all three measures of vigilance.

First, we consider the effect of reproductive status. Both females and non-territorial males in Przewalski's gazelle spent more time vigilant during the rutting than the non-rutting season. The seasonal increase in vigilance probably reflects increased threats associated with rutting as well as environmental changes between seasons [10], [50], [51]. First, seasonal change associated with rutting is probably a driving force for vigilance patterns, as noted in other taxa [23], [30], [31]. During the rutting season, increased aggressive behaviors of dominant males, as a result of changes in physiological status [52], [53], may force subordinate males and females to allocate more time to monitoring conspecifics [8], [15], [54], [55]. Territorial males in Przewalski's gazelle frequently herded and guarded females to keep them inside their rut stands. Territorial males roamed among females and approached them to identify and forcibly mount sexually recipient ones [32], [45]. Aggressive territorial males harass female gazelles and may even cause physical injuries [19], [56], [57]. Therefore, extra scanning by females in rutting season could be directed at territorial males. Non-territorial males are generally subadults or weaker individuals that lost fights to establish dominance at rut stands located along the daily routes taken by females for grazing and drinking [32], [45]. Such failure probably keeps non-territorial males vigilant against aggressive territorial males throughout the rutting season [36]. Non-territorial males typically foraged and traveled far away from territorial males and frequently scanned against potential threats.

In addition to the demands caused by rutting, vigilance may also be influenced by harsh winter conditions, which reduce food quality. Poor foraging during the rutting season forces Przewalski's gazelle to spend more time feeding, as has been noted in other northern hemisphere ungulates [25], [50]. Interestingly, gazelles which faced greater demands on vigilance and foraging during rutting reduced time spent bedding without compromising vigilance. Similar results are found in other studies which investigated tradeoffs between vigilance and other activities within the overall time budget [58], [59]. Whether the reduction in time spent bedding has any consequences on fitness remains to be determined in these gazelles.

We found that territorial males spent the least amount of time on vigilance, which is consistent with results from some ungulate studies [29], [30], [60] but not with others [10], [31]. For males, vigilance patterns may reflect the interplay between species-specific mating strategy and social rank [61], [62]. Unlike impala and captive Père David's deer (*Elaphurus davidianus*), in which dominant males (harem masters) guard fixed rutting territories and are frequently challenged by bachelors [10], [53], territorial males in Przewalski's gazelle spend a large proportion of time herding and guarding roaming harem females. Non-territorial males in Przewalski's gazelle are rarely seen near or inside rut stands to challenge territorial males [36]. Head-on confrontations between territorial males and non-territorial males are thus rarely seen after the establishment of dominance status. In addition, we did not observe any intrusions from other territorial males probably because rut stands are quite scattered (>500 m) in the study area and each harem master only herds his own harem females. Presumably, territorial males benefit from this relatively stable social rank by relaxing vigilance against intruding rivals. Other hypotheses to explain lower vigilance appear less likely. For instance, territorial males during the short rutting season allocate a large proportion of time and energy to rutting activities [50], [63], [64], which may cause a reduction in vigilance against potential threats [30], [65]. However, we found that rutting did not interfere with scanning in territorial males. It is conceivable that territorial males benefit from the vigilance of their harem females while non-territorial males are forced to be more vigilant because they forage in smaller groups. In contrast, we found that group size did not influence vigilance in males.

Low vigilance in territorial males may not unduly influence the risk of being preyed upon. First, any risk is diluted with harem females [13], [54]. Furthermore, both guarding and herding harem females keep territorial males active. During these activities, territorial males can conceivably perceive disturbances detected by more alert females. Nevertheless, we observed that territorial males did not flee away immediately upon detecting an approaching threat, as females did. Territorial males seemed reluctant to leave rut stands. Similar delayed fleeing has also been documented in other mammals and birds [66], [67], [68]. This, together with poor body condition resulting from extensive rutting but less feeding [69], [70], may make territorial males more vulnerable to predation.

As documented by Li et al. [34], we found a negative group size effect on vigilance but in female gazelles only. The sexual difference probably reflects different targets of vigilance in males and females. Any benefits related to collective detection and risk dilution in males is probably compensated by increased monitoring of females in larger groups. The mitigating effect of conspecific monitoring on the group size effect has been predicted and documented in other species [71], [72]. Females probably monitor both males and predators and therefore benefit from the presence of more companions.

What causes the decrease in individual vigilance with group size in females? We argue that the scramble competition hypothesis [15], [54], [55] is unlikely to address this pattern. In spite of low food quality during the rutting season, the alpine meadow where the gazelles forage is homogenous and large, limiting the role of food competition on vigilance [34]. Instead, the observed decline in individual vigilance in large groups probably reflects increased safety caused by the presence of more eyes to detect threats and more bodies to dilute risk [13], [14], [54]. We suggest that risk dilution in larger groups may apply to predation threats as well as threats from territorial males. During the rutting season, territorial males herd and often harass females [32], [45], acting as conspecific threats. The presence of several female companions in larger groups can dilute sexual harassment from the dominant male and allow females to relax vigilance in larger groups. This could be examined in future work by documenting time spent interacting with males for an individual female in groups of different sizes.

Overall, our results indicate that patterns of vigilance in Przewalski's gazelle reflect a complex interplay between reproductive status, social rank, sex, and group size. Similar studies across a broad range of ecological factors will shed more light on factors underlying vigilance in animals and the tradeoff between vigilance and other crucial activities.

## Supporting Information

Table S1 Overall effects of reproductive status, social rank, sex, group size and interactions between factors on percentage time spent scanning in Przewalski's gazelle were tested using a linear model (PROC GLM in SAS).
(DOC)

Table S2 Overall effects of reproductive status, social rank, sex, group size and interactions between factors on mean scan duration in Przewalski's gazelle were tested using a linear model (PROC GLM in SAS).
(DOC)

Table S3 Overall effects of reproductive status, social rank, sex, group size and interactions between factors on scan frequency in Przewalskis' gazelle were tested using a generalized linear model with a negative-binomial error structure and a logarithmic link function (PROC GENMOD in SAS).
(DOC)
