# Supplementary material for: Effects of Reproductive Status, Social Rank, Sex and Group Size on Vigilance Patterns in Przewalski's Gazelle
Source: PLoS One. 2012 Feb 28;7(2):e32607. doi: 10.1371/journal.pone.0032607 (PMC3289666; doi:10.1371/journal.pone.0032607)
Supplement: Table S1 — Overall effects of reproductive status, social rank, sex, group size and interactions between factors on percentage time spent scanning in Przewalski's gazelle were tested using a linear model (PROC GLM in SAS). (DOC) [file pone.0032607.s001.doc]

**Table S1**

|  | R^2^ | df | Type III SS | Mean square | F | t | p |
| --- | --- | --- | --- | --- | --- | --- | --- |
| The final model to test the effects of sex, reproductive status and group size | | | | | | | |
| Model | 0.168 | 4 |  |  | 19.91 |  | <0.001 |
| Error |  | 394 |  |  |  |  |  |
| Total |  | 398 |  |  |  |  |  |
| sex |  | 1 | 0.147 | 0.147 | 17.72 | 4.21 | <0.001 |
| sex × group size |  | 1 | 0.084 | 0.084 | 10.15 | -3.19 | 0.002 |
| reproductive status |  | 1 | 0.055 | 0.055 | 6.61 | -2.57 | 0.011 |
| group size |  | 1 | 0.038 | 0.038 | 4.57 | -2.14 | 0.033 |
| Non-significant effects removed by backward elimination | | | | | | | |
| sex × reproductive status |  | 1 | 0.001 | 0.001 | 0.09 | -0.29 | 0.769 |
| sex × reproductive status × group size |  | 1 | 0.003 | 0.003 | 0.37 | 0.61 | 0.544 |
| reproductive status × group size |  | 1 | 0.011 | 0.011 | 1.26 | -1.12 | 0.262 |
|  |  |  |  |  |  |  |  |
| The final model to test the effect of social rank | | | | | | | |
| Model | 0.211 | 1 |  |  |  |  |  |
| Error |  | 147 |  |  |  |  |  |
| Total |  | 148 |  |  |  |  |  |
| social rank |  | 1 | 0.519 | 0.519 | 39.31 | 6.27 | <0.001 |
| Non-significant effects removed by backward elimination | | | | | | | |
| group size |  | 1 | 0.003 | 0.003 | 0.24 | 0.49 | 0.625 |
| social rank × group size |  | 1 | 0.004 | 0.004 | 0.29 | -0.54 | 0.590 |
