# Supplementary material for: Effects of Reproductive Status, Social Rank, Sex and Group Size on Vigilance Patterns in Przewalski's Gazelle
Source: PLoS One. 2012 Feb 28;7(2):e32607. doi: 10.1371/journal.pone.0032607 (PMC3289666; doi:10.1371/journal.pone.0032607)
Supplement: Table S2 — Overall effects of reproductive status, social rank, sex, group size and interactions between factors on mean scan duration in Przewalski's gazelle were tested using a linear model (PROC GLM in SAS). (DOC) [file pone.0032607.s002.doc]

**Table S2**

|  | R^2^ | df | Type III SS | Mean square | F | t | p |
| --- | --- | --- | --- | --- | --- | --- | --- |
| The final model to test the effects of sex, reproductive status and group size | | | | | | | |
| Model | 0.053 | 2 |  |  | 3.14 |  | 0.003 |
| Error |  | 396 |  |  |  |  |  |
| Total |  | 398 |  |  |  |  |  |
| sex |  | 1 | 1.188 | 1.188 | 6.98 | -2.64 | 0.009 |
| group size |  | 1 | 0.695 | 0.695 | 4.08 | -2.02 | 0.044 |
| Non-significant effects removed by backward elimination | | | | | | | |
| sex × group size |  | 1 | 0.013 | 0.013 | 0.08 | -0.28 | 0.779 |
| sex × reproductive status × group size |  | 1 | 0.020 | 0.020 | 0.12 | 0.34 | 0.734 |
| reproductive status × group size |  | 1 | 0.090 | 0.090 | 0.52 | -0.72 | 0.469 |
| reproductive status |  | 1 | 0.336 | 0.336 | 1.97 | -1.41 | 0.161 |
| sex × reproductive status |  | 1 | 0.410 | 0.410 | 2.41 | 1.55 | 0.121 |
|  |  |  |  |  |  |  |  |
| The final model to test the effect of social rank (no significant effects) | | | | | | | |
| social rank × group size |  | 1 | 0.020 | 0.020 | 0.12 | 0.35 | 0.726 |
| group size |  | 1 | 0.025 | 0.025 | 0.15 | 0.35 | 0.698 |
| social rank |  | 1 | 0.078 | 0.078 | 0.49 | 0.70 | 0.487 |
