# Supplementary material for: Effects of Reproductive Status, Social Rank, Sex and Group Size on Vigilance Patterns in Przewalski's Gazelle
Source: PLoS One. 2012 Feb 28;7(2):e32607. doi: 10.1371/journal.pone.0032607 (PMC3289666; doi:10.1371/journal.pone.0032607)
Supplement: Table S3 — Overall effects of reproductive status, social rank, sex, group size and interactions between factors on scan frequency in Przewalskis' gazelle were tested using a generalized linear model with a negative-binomial error structure and a logarithmic link function (PROC GENMOD in SAS). (DOC) [file pone.0032607.s003.doc]

**Table S3**

|  | df | Value/df | χ^2^ | p |
| --- | --- | --- | --- | --- |
| The final model to test the effects of sex, reproductive status and group size | | | | |
| Deviance | 396 | 1.031 |  |  |
| Pearson χ^2^ | 396 | 1.048 |  |  |
| sex | 1 |  | 134.63 | <0.001 |
| sex × group size | 1 |  | 27.15 | <0.001 |
| Non-significant effects removed by backward elimination | | | | |
| group size | 1 |  | 0.00 | 0.978 |
| sex × reproductive status × group size | 1 |  | 0.17 | 0.682 |
| reproductive status | 1 |  | 0.96 | 0.327 |
| sex × reproductive status | 1 |  | 1.69 | 0.194 |
| reproductive status × group size | 1 |  | 2.03 | 0.155 |
|  |  |  |  |  |
| The final model to test the effect of social rank | | | | |
| Deviance | 147 | 1.098 |  |  |
| Pearson χ^2^ | 147 | 0.878 |  |  |
| social rank | 1 |  | 72.1 | <0.001 |
| Non-significant effects removed by backward elimination | | | | |
| social rank × group size | 1 |  | 0.01 | 0.929 |
| group size | 1 |  | 0.02 | 0.892 |
